# Plasma exosomal microRNAs are non-invasive biomarkers of moyamoya disease: A pilot study

**DOI:** 10.1016/j.clinsp.2023.100247

**Published:** 2023-07-05

**Authors:** Da Huang, Hui Qi, Hongchun Yang, Meng Chen

**Affiliations:** Department of Neurosurgery, Peking University Shenzhen Hospital, Shenzhen, China

**Keywords:** Moyamoya disease, Exosomal microRNA, Actin cytoskeleton signalling pathway, Plasma, Biomarkers

## Abstract

**Background:**

As a progressive cerebrovascular disease, Moyamoya Disease (MMD) is a common cause of stroke in children and adults. However, the early biomarkers and pathogenesis of MMD remain poorly understood.

**Methods and material:**

This study was conducted using plasma exosome samples from MMD patients. Next-generation high-throughput sequencing, real-time quantitative PCR, gene ontology analysis, and Kyoto Encyclopaedia of Genes and Genomes pathway analysis of ideal exosomal miRNAs that could be used as potential biomarkers of MMD were performed. The area under the Receiver Operating Characteristic (ROC) curve was used to evaluate the sensitivity and specificity of biomarkers for predicting events.

**Results:**

Exosomes were successfully isolated and miRNA-sequence analysis yielded 1,002 differentially expressed miRNAs. Functional analysis revealed that they were mainly enriched in axon guidance, regulation of the actin cytoskeleton and the MAPK signaling pathway. Furthermore, 10 miRNAs (miR-1306-5p, miR-196b-5p, miR-19a-3p, miR-22-3p, miR-320b, miR-34a-5p, miR-485-3p, miR-489-3p, miR-501-3p, and miR-487-3p) were found to be associated with the most sensitive and specific pathways for MMD prediction.

**Conclusions:**

Several plasma secretory miRNAs closely related to the development of MMD have been identified, which can be used as biomarkers of MMD and contribute to differentiating MMD from non-MMD patients before digital subtraction angiography.

## Highlights

• miRNA-seq analysis obtained 1,002 differentially expressed miRNAs.
• Differentially expressed miRNAs were mainly enriched in axon guidance, regulation of the actin cytoskeleton and the MAPK signaling pathway.
• 10 miRNAs (miR-1306-5p, miR-196b-5p, miR-19a-3p, miR-22-3p, miR-320b, miR-34a-5p, miR-485-3p, miR-489-3p, miR-501-3p, and miR-487-3p) were found to be associated with the most sensitive and specific pathways for MMD prediction.

## Introduction

Moyamoya Disease (MMD) is a cerebrovascular disease with unknown etiology and progressive, occlusive, and abnormal collateral vascular networks. It was named “Moyamoya disease” because the abnormal network of vessels at the skull base resembles “smoke” in cerebral angiography.

MMD is highly prevalent in Northeast Asia, especially in Japan, Korea, and China, with some familial inclination.2] The incidence and prevalence of MMD in China are 0.59 and 1.01 per 100,000 person-years, respectively.^1^ MMD maintains a classical pattern of bimodal age distribution, with the first peak occurring at the age of five and the second at the age of 40. The incidence rate in women is higher than that in men (female-to-male ratio is 1.12).^1^ The clinical symptoms of patients are complex and varied, including cognitive impairment, epilepsy, involuntary movements or headache, the most common of which is cerebral ischemia, which can manifest as transient ischemic attacks, reversible ischemic neurologic deficit, or cerebral infarction.

The pathogenesis of MMD is not fully understood and treatment options are quite limited. Recent studies suggest that the pathogenesis of MMD may involve angiogenesis, genetic factors, and immune inflammation.^2^ However, the specific mechanism is still unknown.^3^ Although the related genetic background or related genes or similar differ significantly among different races, MMD is associated with the class I and class II genes of the Human Leukocyte Antigen (HLA).^4^ When smooth muscle proliferates, macrophages and T-lymphocytes infiltrate the intimal surface, resulting in intimal hyperplasia and lumen stenosis, which may be the primary inflammatory mechanism in MMD.^5^ With the maturation of multimodal Three-Dimensional (3D) angiography technology, the understanding of MMD and the standardization of diagnostic standards are improving, which has increased the detection rate of MMD worldwide year by year.

The treatment of MMD mainly revolves around surgical intervention to improve blood circulation in the affected region by performing direct or indirect revascularization surgeries.^6^ Nevertheless, a clear conclusion has not been reached on the pathogenesis and evolution of MMD, and there is still a lack of effective biomarkers and molecularly targeted therapies. Recently, Pinard et al. ^7^ found that rare variants in ANO1 (encoding a calcium-activated chloride channel) predispose patients to moyamoya disease. Ring Finger protein 213 (RNF213) has recently been identified as a susceptibility gene for MMD, whose apoptosis-inducing function may be negatively regulated by its ubiquitin ligase.^8^, ^9^, ^10^ However, mice lacking the RNF213 gene did not spontaneously develop MMD, indicating that the loss of function of RNF213 could not fully induce MMD.^11^ Therefore, although RNF213 may be a pathogenic factor in MMD, its specific mechanism in pathogenesis is still unclear. There is an urgent need to develop ideal and effective biomarkers to be used as diagnostic, therapeutic, and prognostic agents in MMD.

MicroRNAs (miRNAs) are single-stranded RNA molecules, approximately 21‒23 nucleotides in length, that participated in many biological processes, including cell proliferation and differentiation, cell migration, and disease progression. In many cases, miRNAs are considered to be minimally invasive markers for the early detection of diseases with high specificity.^12^ In particular, miRNAs in blood or specific fluid components are candidates for improving disease diagnosis, including disease course judgment. Exosomes are extracellular membrane vesicles 30‒150 nm in diameter that are present in almost all biological fluids and are rich in mRNA, miRNA, and other non-coding RNAs. When exosomes circulate, these RNAs can be carried to neighboring or distant cells, and subsequently regulate recipient cells to function in genetic exchange between cells.^13^ Studies have found that exosomal miRNAs derived from the cerebrospinal fluid of MMD patients may serve as diagnostic biomarkers for diagnosis.^14^

Therefore, this study analyzed the miRNAs expressed in exosomes from plasma samples from MMD patients and healthy individuals via Next-Generation Sequencing (NGS). By quantifying the expression profile of exosomal miRNAs, the miRNAs statistically correlated with MMD diagnosis were identified to determine pathways that potentially mediate the pathogenesis and biomarkers of MMD, with the hope of mining for ideal biomarkers for MMD diagnosis, treatment, and prognosis.

## Methods

### Patients and sample collection

Nine patients diagnosed with MMD by digital subtraction angiography were enrolled at Peking University Shenzhen Hospital from December 2020 to March 2021. Ten patients with suspected MMD but excluded by digital subtraction angiography (non-MMD patients) served (non-MMD patients) as the control group (NC). All subjects signed an informed agreement. For peripheral blood collection, 3 mL of peripheral blood from enrolled participants was collected into 5 mL K2EDTA Vacutainer tubes (Junnuo, Chengwu, China). The study was approved by the medical ethics committee of Peking University Shenzhen Hospital. All procedures were ethically guided by the principles of the 1964 Declaration of Helsinki and its 2013 amendment.

### Exosome isolation and miRNA extraction

The plasma supernatant was collected and centrifuged at 3,500g at 4°C for 10 min, and then the supernatant was centrifuged at 12,000g at 4°C for 10 min. The precipitate was discarded, and the supernatant was collected for exosome extraction. Exosomes were extracted based on ExoQuick precipitation using an ExoQuick precipitation system (System Biosciences Inc., CA, United States).

### Identification of exosomes

Transmission Electron Microscopy (TEM), Nanoparticle Tracking Analysis (NTA), and Western blot were used to identify exosome features.

The biological morphology of exosomes was observed by negative staining with TEM. In brief, purified exosomes were fixed with 2% Paraformaldehyde (PFA) for 5 min at room temperature. The extracted exosomes were diluted 1:20, and then 10 µL was added to a copper net under an electron microscope and heated in an oven at 65°C for 30 min. After drying, the exosomes were labeled with 1% Uranyl Acetate (UA) phosphotungstic acid and dried for 10 min at room temperature. The size and characteristics of the exosomes were observed under a transmission electron microscope (Tecnai G2 Spirit BioTWIN; FEI Company).

Nanoparticle tracking analysis (NTA, Particle Metrix, Germany) was used to identify the exosomes. The NTA software ZetaView 8.04.02 SP2 (Particle Metrix, Germany) was used for data acquisition and processing according to the manufacturer's instructions. The supernatant was filtered through a 0.45 μm PBS filter, mixed with 0.5M EDTA, pH 8.0 (Life Technologies, USA), and then adjusted to Ph 4.2. The solution was centrifuged at 300g for 10 min at 4°C, the supernatant was collected, and the pH was adjusted to 7.0. The ambient temperature was set at 24°C, while background extraction and automatic settings were applied for the minimum expected particle size, minimum track length, and blur. The samples were diluted 1:50 following sterile filtration with vesicle-free DPBS. Each experiment was repeated three times and performed in triplicate.

Using Western blot to detect marker proteins in exosomes. Exosomes were dissolved in RIPA buffer (ASPEN, Wuhan, China), and the protein concentrations were determined using a BCA Protein Assay Kit (ASPEN, Wuhan, China). Protein extracts were separated by 10% sodium dodecyl Sulfate-Polyacrylamide Gel Electrophoresis (SDS–PAGE) and transferred onto a Polyvinylidene Difluoride (PVDF) membrane (Millipore, MA, United States). The membranes were incubated with CD63 (Abcam, ab217345) and CD81 (Abcam, ab109201) primary antibodies at 4°C overnight after blocking with 5% BSA. Next, the corresponding secondary antibodies were incubated at room temperature for 1h. The protein bands were visualized using Immobilon ECL Ultra Western HRP Substrate (ASPEN, Wuhan, China). A Tanon-5500 Chemiluminescent Imaging System (Tanon Science & Technology, Shanghai, China) was used for visualization imaging. RNA library construction and sequencing

TRIzol reagent (Invitrogen Life Technologies, Carlsbad, CA) was used to extract total RNA from the exosome pellets following the manufacturer's instructions. The total RNA concentration and quality were quantified using a Qubit3 Fluorometer (Thermo Fisher Scientific, MA, United States) and an Agilent Bioanalyzer 2100 (Agilent Technologies, CA, United States).

The small RNA library was constructed according to the QIAseq miRNA Library kit (QIAGEN, Germany). Approximately 100 ng of total RNA was used to prepare the miRNA library and supplemented with water to 20 μL. Then, reverse transcription of the sample was performed to obtain cDNA. QIAseq miRNA NGS beads were used to wash cDNA several times according to the manufacturer's instructions. The quality control of the small RNA library (total RNA > 0.05ug) was analyzed using an Agilent Bioanalyzer 2100. The qualifying small RNA libraries were sequenced on a HiSeq 2500 (Illumina, San Diego, USA).

### Data processing and bioinformatics analysis

Raw data from the small RNA sequencing were processed to estimate microRNA expression. The authors calculated the number of reads derived from a gene Per Million Reads (RPM) to eliminate the effect of sequencing depth on the read count. miRNAs with read counts greater than or equal to 10 were considered to be expressed, while read counts of less than 10 were considered to indicate no expression. The authors performed PCA on the RPM values of the screened genes to show the grouping information of each sample under different experimental conditions. Pearson correlation coefficients between biological replicates were calculated to assess sample reliability.

MiRNAs with an adjusted p-value < 0.01 and fold change > 2 determined by edgeR were considered differentially expressed. The miRNA targets were predicted using the prediction website TargetScan (http://www.targetscan.org/vert_71/). Gene Ontology (GO) enrichment analysis was performed via topGO, and Kyoto Encyclopaedia of Genes and Genomes (KEGG) pathway analysis was performed using clusterProfiler (kobas2.0-20150126).

### Real-time quantitative PCR (RT-qPCR)

The miRNA expression levels were assessed by RT-qPCR. Total RNA was reverse transcribed to cDNA using the PrimeScript First Strand cDNA Synthesis Kit (Takara, Beijing, China). RT-qPCR was carried out using a 2 × SYBR green qPCR mix (Takara, Beijing, China) and an ABI 7900HT sequence system (Thermo Fisher Scientific, Inc.). The reactions were incubated at 94°C for 3 min, followed by 40 cycles at 95°C for 15s and 62°C for 40s. The primer sequences for the four miRNAs are shown in Supplementary Table 1. U6 was used as an internal control for miRNAs. Statistical analyses of the results were performed using the 2^−∆∆CT^ relative quantification method.

### Statistical analysis

Student's *t*-test was applied to the RT-qPCR analysis results to compare the different groups using GraphPad Prism 5.0. ROC curves and AUC values were determined using the edgeR package. GO and KEGG pathway analyses were assessed by Fisher's exact test to identify significant results using the edgeR package; p-values < 0.05 were significant.

## Results

### The clinical information of the sample

**Fig. 1:**
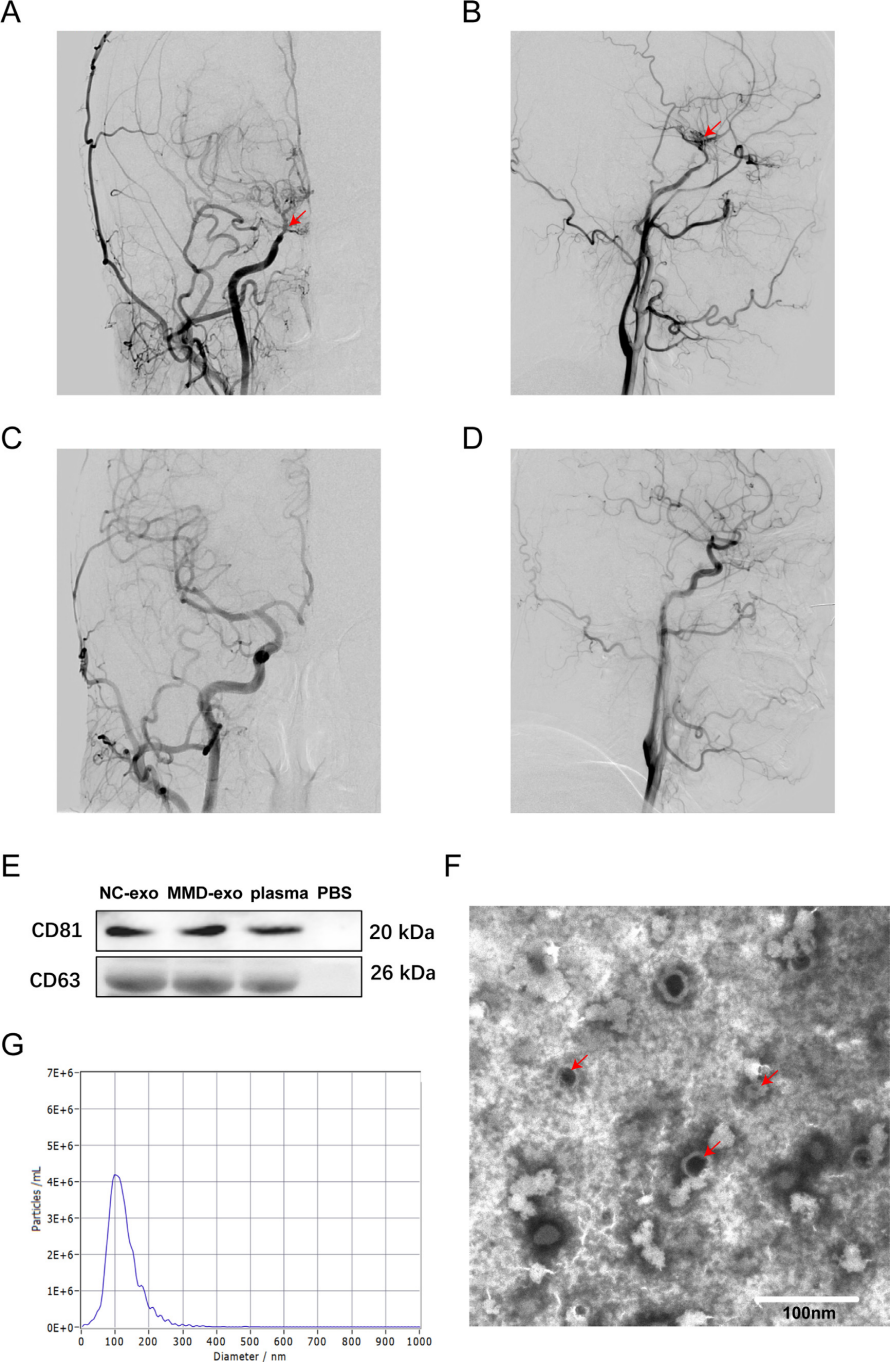
Digital subtraction angiography (DSA) images and characterization of exosomes in the plasma of moyamoya disease patients. (A‒B) Digital Subtraction Angiography (DSA) images of moyamoya disease patients. The arrows on the pictures show the narrowing/stenosis of the carotid artery that causes moyamoya disease. (C‒D) Digital subtraction angiography (DSA) images of nonmoyamoya disease patients. A and C are standard anterior positions, B, D are standard lateral positions. (E) The protein levels of CD81 and CD63 in exosomes were analysed by western blotting. (F) Transmission electron microscopy image of a mixture of NC and MMD exosomes. The arrows on the pictures show exosomes. Scale bar, 100 nm. (G) Size distribution of exosomes determined by nanoparticle tracking analysis. NC-exo, Exosomes from Non-MMD patients; MMD-exo, Exosomes from MMD patients.

The clinical information of the sample is shown in Table 1. Based on whether smoke-like changes were observed in the brain during the digital subtraction cerebrovascular angiography examination or similar, the authors could distinguish patients with or without MMD. For example, Fig. 1 A and B show a patient with the same hemorrhagic moyamoya disease (bilateral), characterized by cerebral hemorrhage, occlusion of the end of the internal carotid artery on the right side, and smoke vessel formation. Fig. 1 C and D are from the same non-MMD patient, showing normal cerebral blood vessels.

**Table 1:** Clinical information of the samples.

	NC	MMD	*p*-value
**Male sex**	5(50)	5(56%)	0.967
**Age**	46.1 ± 6.4	48.9 ± 12.1	0.603
**WBC (× 10^9^)**	6.08 ± 1.24	7.23 ± 1.37	0.169
**RBC (× 10^9^)**	4.45 ± 0.32	4.45 ± 0.41	0.967
**Hb (mmoL/L)**	133 ± 7.1	132 ± 13	0.791
**Glu (mmoL/L)**	5.31 ± 0.62	5.21 ± 0.65	0.729
**K^+^ (mmoL/L)**	4.02 ± 0.21	3.95 ± 0.21	0.456
**Na^+^ (mmoL/L)**	139.7 ± 3.4	141.1 ± 2.5	0.306
**ALT (U/L)**	19.5 ± 6.6	24.45 ± 15.18	0.364
**TB (µmoL/L)**	9.38 ± 3.03	8.58 ± 3.01	0.561
**DB (µmoL/L)**	1.92 ± 0.19	1.75 ± 0.39	0.232
**TC (mg/DL)**	3.63 ± 1.03	3.88 ± 1.57	0.678
**TG (mg/DL)**	1.41 ± 0.69	1.54 ± 0.87	0.737
**HDL (mg/DL)**	0.99 ± 0.07	0.97 ± 0.27	0.85
**LDL (mg/DL)**	2.88 ± 0.63	3.02 ± 0.77	0.662

WBC, White Blood Cell; RBC, Red Blood Cell; Hb, Hemoglobin; Glu, Glucose; ALT, Alanine Transaminase; TB, Tuberculosis; DB, Direct Bilirubin; TC, Total Cholesterol; TG, Triglyceride; HDL, High-Density Lipoprotein; LDL, Low Density Lipoprotein; NC, Healthy individuals; MMD, Moyamoya Disease Patients.

### Characterization of exosomes obtained from MMD patient plasma

To characterize exosomes derived from MMD patient plasma, TEM and NTA were used to characterize exosome diameters and CD63 and CD81, protein markers of exosomes, were assessed in exosomes of NC, MMD, and plasma by Western blotting analysis. The authors assessed the exosome protein markers CD63 and CD81 in the exosomes of all group pairs, which confirmed that we had indeed isolated exosomes (Fig. 1E). TEM showed a typical rounded morphology with a sagged double membrane (Fig. 1F). NTA further confirmed that the exosomes isolated from MMD patients and healthy individuals were 30‒150 nm in diameter (Fig. 1G). These results indicated that exosomes were successfully purified from all plasma samples.

### miRNA sequencing and data analysis

**Fig. 2:**
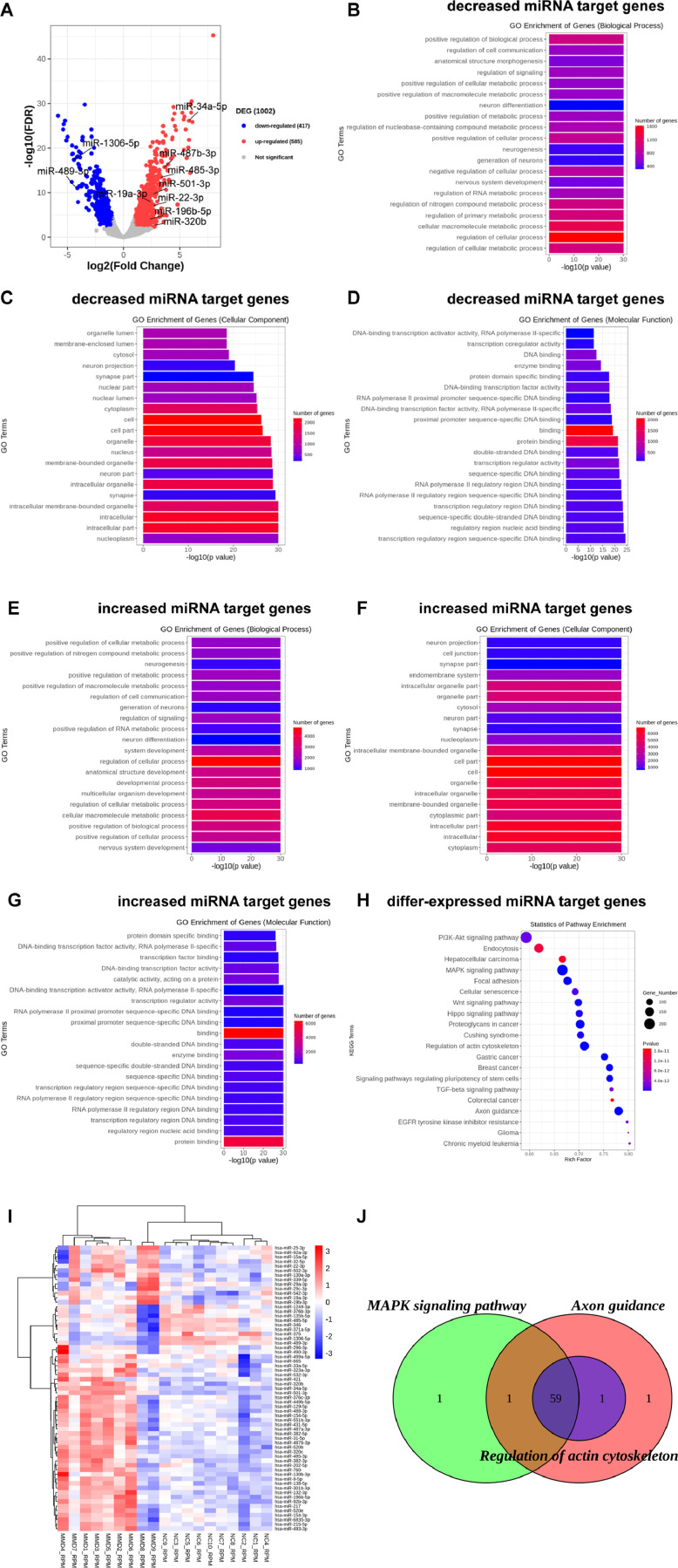
miRNA expression analysis and enrichment analysis of target genes of significantly differentially expressed miRNAs. (A) Volcano plot of significantly differentially expressed miRNAs between moyamoya disease patients and the control group. (B‒D) GO enrichment analysis of target genes of decreased expressed miRNAs in biological process (B), cellular components (C), and molecular function (D). (E‒G) GO enrichment analysis of target genes of increased expressed miRNAs in biological process (E), cellular components (F), and molecular function (G). (H) KEGG enrichment analysis of target genes of differentially expressed miRNAs. (I) Heatmap of differentially expressed miRNAs involved in axon guidance, regulation of the actin cytoskeleton and the MAPK signaling pathway. (J) Venn diagram of differentially expressed miRNAs involved in axon guidance, regulation of the actin cytoskeleton and the MAPK signalling pathway. NC, Non-MMD patients; MMD, Moyamoya disease.

To obtain differences in miRNA expression profiles between MMD patients and non-MMD patients, plasma exosomal RNA samples were used for miRNA sequencing. Raw data were filtered to obtain 287,021,585 bp clean data (Supplementary Table 2). Principal component analysis and correlation analysis were performed to demonstrate the validity of the grouped samples and data (Supplementary Fig. 1). Differentially expressed miRNAs were screened based on the adjusted p-value < 0.01 and fold change > 2. A total of 1,002 differentially expressed miRNAs were identified, including 585 upregulated and 417 downregulated miRNAs (Fig. 2A).

GO and KEGG enrichment analyses were performed on these differentially expressed miRNAs. The GO results indicated that the decreased expressed miRNAs were primarily associated with biological process terms, including regulation of the cellular metabolic process, regulation of the cellular process, and cellular macromolecule metabolic process (Fig. 2B). They were also enriched in cellular component terms, including nucleoplasm, and intracellular part (Fig. 2C), and in molecular function terms, including transcription regulatory region sequence-specific DNA binding and regulatory region nucleic acid binding (Fig. 2D). In contrast, miRNAs with upregulated expression were primarily associated with biological process terms, including nervous system development, and positive regulation of the cellular process, positive regulation of biological process (Fig. 2E); cellular component terms, including cytoplasm, intracellular, and intracellular part (Fig. 2F), and molecular function terms, including protein binding and regulatory region nucleic acid binding (Fig. 2G). Moreover, KEGG pathways of their target genes were mainly involved in axon guidance, regulation of the actin cytoskeleton, and the MAPK signaling pathway (Fig. 2H), and further statistical analysis revealed 63 exosomal miRNAs involved in these three signaling pathways (Fig. 2I). Among them, 59 exosomal miRNAs with different target genes were associated with all three pathways at the same time, especially with the regulation of the actin cytoskeleton (Fig. 2J).

### Prognosis of potential biomarkers as determined by the receiver operating characteristic curve and area under the curve

**Fig. 3:**
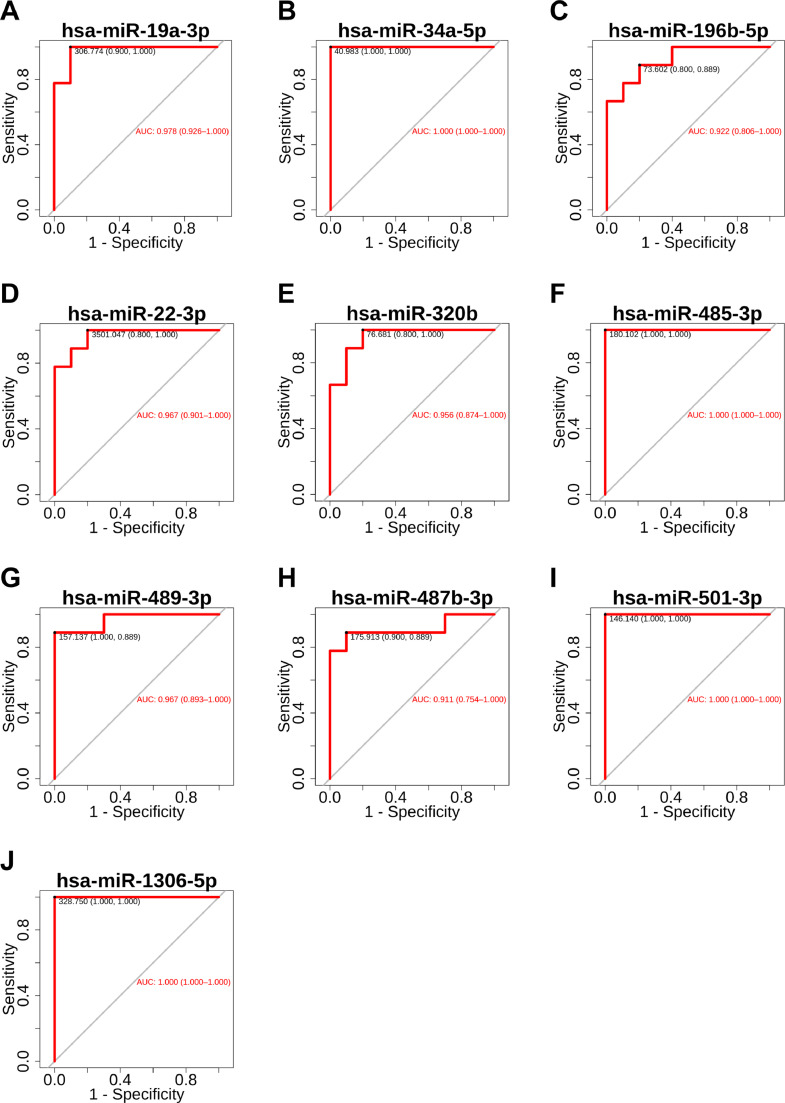
AUC of the receiver operating characteristic (ROC) curve analysis. Ten miRNAs (miR-1306-5p, miR-196b-5p, miR-19a-3p, miR-22-3p, miR-320b, miR-34a-5p, miR-485-3p, miR-487b-3p, miR-489-3p, miR-501-3p) with both the sensitivity and specificity above 0.8.

To assess the diagnostic values of the above 63 miRNAs involved in axon guidance, regulation of the actin cytoskeleton and the MAPK signaling pathway, the AUC of ROC was carried out to differentiate diseased individuals. The analysis revealed that 10 miRNAs (miR-1306-5p, miR-196b-5p, miR-19a-3p, miR-22-3p, miR-320b, miR-34a-5p, miR-485-3p, miR-489-3p, miR-501-3p, and miR-487b-3p) had AUC value higher than 0.9, sensitivity and specificity higher than 0.8, which could clearly distinguished MMD patients from non-MMD patients (Fig. 3).

**Fig. 4:**
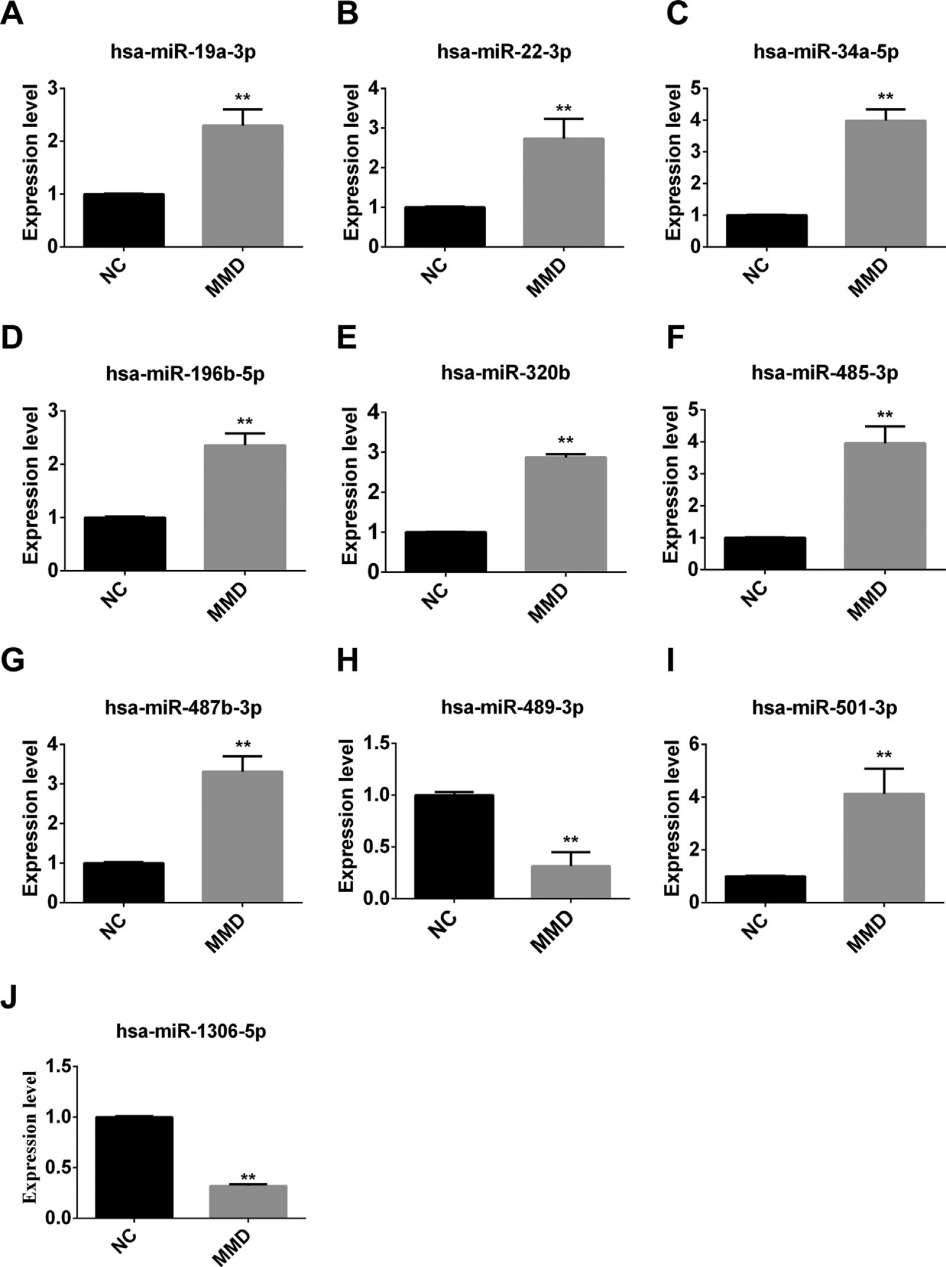
The levels of miRNAs were analysed by RT-QPCR. The different expression levels of ten miRNAs (miR-1306-5p, miR-196b-5p, miR-19a-3p, miR-22-3p, miR-320b, miR-34a-5p, miR-485-3p, miR-487b-3p, miR-489-3p, miR-501-3p) between 9 moyamoya disease patients and 10 healthy individuals. NC, Non-MMD patients; MMD, Moyamoya Disease. The data represent the mean ± S.E.M. of three independent experiments. *p < 0.05, **p < 0.01.

To further explore the expression of these exosomal miRNAs as potential biomarkers of MMD, RT-qPCR was performed to randomly determine the levels of these exosomal miRNAs extracted from the plasma samples of MMD patients and non-MMD patients. The 10 microRNAs showed the same expression patterns as those obtained from the high-throughput sequencing analysis. MiR-34a-5p, miR-19a-3p, miR-22-3p, miR-196b-5p, miR-320b, miR-485-3p, miR-487b-3p, and miR-501-3p were upregulated, and miR-489-3p and hsa-miR-1306-5p were downregulated (Fig. 4).

## Discussion

MMD is a disease of the central nervous system, and the molecular changes in cerebrospinal fluid may reflect the molecular conditions of the central nervous system.^15^ However, cerebrospinal fluid cannot be extracted from patients in certain age groups and may have certain restrictions in its application.^16^^,^^17^ Peripheral blood is a readily available and widely targeted biological fluid and its biomarkers represent a novel and valuable approach to performing humoral diagnostics. By mining and validating ideal miRNA analysis from peripheral blood exosomes, the authors can develop a non-invasive rapid detection kit for the early diagnosis, treatment, and prognosis of MMD. In this study, miRNAs significantly associated with MMD were identified by isolating exosomes from patient plasma and performing miRNA sequencing. To the best of our knowledge, this is the first study to perform comprehensive high-throughput sequencing analysis of all exosomal miRNA profiles extracted from plasma.

A number of miRNAs may serve as biomarkers of clinical MMD, and their possible involvement in the pathogenesis of MMD is illustrated by bioinformatic analysis and experimental validation. In this study, the authors identified a total of 1,002 differentially expressed exosomal miRNAs, and the top three major signaling pathways enriched for the target genes of these differentially expressed miRNAs were axon guidance, regulation of the actin cytoskeleton, and the MAPK signaling pathway. A proteomic analysis of the serum exosomes from MMD patients in one study showed that 859 shared proteins were detected in serum exosomes from patients with ischaemic and hemorrhagic MMD, 231 of which were different from those in the healthy controls, and that protein imbalances and actin dynamics disorder related to cell growth and maintenance were closely associated with MMD.^18^ Ren et al.^19^ found that both MMD patients and Neo1 (neogenin) mutant mice exhibited altered gene expression in their cortex in proteins critical for axon guidance, suggesting that the Neo1-related axon guidance pathway may play important roles in regulating MMD-like vasculopathy. There is also research indicating that the MAPK signaling pathway is active in the pathogenesis of MMD.^20^ Ota et al. identified some miRNAs that may be related to the etiology and pathophysiology of moyamoya disease by analyzing the expression levels of microRNAs derived from extracellular vesicles in the cerebrospinal fluid of patients with moyamoya disease.^21^ Dai et al. identified 94 differentially expressed miRNAs in serum samples from 10 patients and 10 controls through genome-wide miRNA array analysis.^22^ These findings further supported the present results that plasma exosomal miRNAs are potential biomarkers, that contribute to the study of MMD.

Moreover, the authors performed AUC of the ROC curve analysis of miRNAs and found that miR-1306-5p, miR-196b-5p, miR-19a-3p, miR-22-3p, miR-320b, miR-34a-5p, miR-485-3p, miR-489-3p, miR-487b-3p, and miR-501-3p had high sensitivity and specificity for predicting MMD. The involvement of these miRNAs in cerebrovascular disease has been reported by a number of researchers. For example, transfection of miR-1306-5p mimics eliminated the inhibitory effect of SNHG7-003 overexpression on the proliferation and migration of vascular smooth muscle cells.^23^ MiR-34a-5p participates in the autophagy of human coronary artery endothelial cells induced by chronic intermittent hypoxia through the Bcl-2/Beclin1 signal transduction pathway.^24^ In addition, the authors predicted that miR-34a-5p would interact with RNF213 in the TargetScan database. Inhibitors of miR-485-3p can promote the proliferation of human microvascular endothelial cells under hypoxic conditions.^25^ High expression of exosomal miR-501-3p promotes vascular sclerosis.^26^ miR-22-3p can regulate human artery vascular smooth muscle cell proliferation and migration by targeting HMGB1, and may be a therapeutic target for the treatment of human arteriosclerosis obliterans.^27^ Serum miR-19a-3p is considered a diagnostic biomarker for asymptomatic carotid artery stenosis and a promising predictor of cerebral ischemia events.^28^ miR-320b is a specific serum marker for carotid atherosclerosis and vulnerable plaques, which could be used to assist in the diagnosis of cerebrovascular diseases.^29^ These previous results support the present findings that imbalances in miRNA expression have a substantial impact on MMD, but further validation by higher-level data and mechanism-based analysis is still needed.

In addition, miR-196b-5p, miR-487b-3p, and miR-489-3p were first reported to be related to MMD. Based on the mechanisms of action of these miRNAs in other diseases reported in the literature, the authors speculate that these miRNAs may play a pathological role in moyamoya disease by participating in angiogenesis^30^ and immune inflammation.^31^ However, further research is needed on its mechanism of action in moyamoya disease.

## Conclusions

In summary, the authors generated a comprehensive miRNA expression profile of plasma exosomes in MMD patients by high-throughput sequencing and found that the regulation of the actin cytoskeleton pathway may be primarily involved in the pathogenesis of MMD. Moreover, the current study identified some exosomal miRNAs that could be explored in future studies as potential biological targets for the diagnosis of MMD and mediating the pathogenesis, in an attempt to improve the long-term benefits for patients. However, a limitation of this study is that the number of patients enrolled was small. Furthermore, the molecular mechanisms of these miRNAs may relate to more complex biological pathways in MMD and require more in-depth exploration in the future.

## Data availability statements

The data that support the findings of this study are available from the corresponding author upon reasonable request. The raw data files of high-throughput sequencing are available in the zenodo repository, DOI:10.5281/zenodo.5800422.

## Consent for publication

All participants in the study agreed to be published.

## Ethical statement

The study was approved by the medical ethics committee of Peking University Shenzhen Hospital. All subjects signed an informed agreement.

## Funding

This work was supported by Shenzhen Municipal Health and Family Planning System Scientific Research Project n SZXJ2017052.

## CRediT authorship contribution statement

**Da Huang:** Conceptualization, Methodology, Writing – original draft. **Hui Qi:** Methodology, Formal analysis, Writing – original draft. **Hongchun Yang:** Investigation, Visualization. **Meng Chen:** Software, Formal analysis.

## Declaration of Competing Interest

The authors declare no conflicts of interest.

## Appendix Supplementary materials

Image, application 1

Image, application 2
