# Supplementary material for: Plasma exosomal microRNAs are non-invasive biomarkers of moyamoya disease: A pilot study
Source: Clinics (Sao Paulo). 2023 Jul 5;78:100247. doi: 10.1016/j.clinsp.2023.100247 (PMC10344806; doi:10.1016/j.clinsp.2023.100247)
Supplement: Supplementary file 1 [file mmc1.docx]

**CLINICS-D-23-00096_ Supplementary Material**

**Supplement Table 1** Information of validated miRNAs.

| **miRNA** | **Primers (5’‒ 3’)** |
| --- | --- |
| hsa-miR-1306-5p | CCACCTCCCCTGCAAACGTCCA |
| hsa-miR-22-3p | AAGCTGCCAGTTGAAGAACTGT |
| hsa-miR-34a-5p | TGGCAGTGTCTTAGCTGGTTGT |
| hsa-miR-489-3p | GTGACATCACATATACGGCAGC |
| hsa-miR-501-3p | AATGCACCCGGGCAAGGATTCT |
| has-miR-196b-5p | TAGGTAGTTTCCTGTTGTTGGG |
| has-miR-19a-3p | TGTGCAAATCTATGCAAAACTGA |
| has-miR-320b | AAAAGCTGGGTTGAGAGGGCAA |
| has-miR-485-3p | GTCATACACGGCTCTCCTCTCT |
| has-miR-487b-3p | AATCGTACAGGGTCATCCACTT |

**Supplement Table 2** Sequencing data output statistics and quality control.

| **Sample Name** | **Raw Reads** | **Clean Reads** | **Q20** | **Q30** | **GC%** |
| --- | --- | --- | --- | --- | --- |
| MMD1 | 13394601 | 11470036 | 0.994038 | 0.979452 | 0.541002 |
| MMD2 | 16789838 | 15597279 | 0.993253 | 0.977721 | 0.543984 |
| MMD3 | 19767133 | 14471822 | 0.993802 | 0.979251 | 0.541744 |
| MMD4 | 10192610 | 8096998 | 0.994197 | 0.979526 | 0.552548 |
| MMD5 | 13430381 | 11318666 | 0.993618 | 0.978735 | 0.542509 |
| MMD6 | 19573465 | 17755102 | 0.994607 | 0.98019 | 0.538964 |
| MMD7 | 20702651 | 17529882 | 0.99429 | 0.980239 | 0.527753 |
| MMD8 | 18527733 | 17109638 | 0.99283 | 0.977045 | 0.502724 |
| MMD9 | 21319758 | 20473411 | 0.993715 | 0.978213 | 0.487527 |
| NC1 | 12058148 | 11396788 | 0.984477 | 0.949508 | 0.552691 |
| NC2 | 37649858 | 36311886 | 0.990424 | 0.966974 | 0.548631 |
| NC3 | 21111513 | 18796751 | 0.989343 | 0.965364 | 0.566005 |
| NC4 | 35556828 | 29895610 | 0.986185 | 0.954861 | 0.548253 |
| NC5 | 11792452 | 11097666 | 0.985199 | 0.951597 | 0.559581 |
| NC6 | 15052720 | 4073973 | 0.992414 | 0.974378 | 0.585476 |
| NC7 | 11093372 | 5542318 | 0.992088 | 0.973208 | 0.580912 |
| NC8 | 10618213 | 7722455 | 0.991523 | 0.972106 | 0.588273 |
| NC9 | 11282238 | 7778548 | 0.992302 | 0.974028 | 0.582235 |
| NC10 | 30780243 | 20582756 | 0.992305 | 0.974105 | 0.583265 |

**Supplemental Figure 1** Principal component diagram (A) and correlation analysis diagram (B) of samples. NC, Non-MMD patients; MMD, Moyamoya Disease.

**
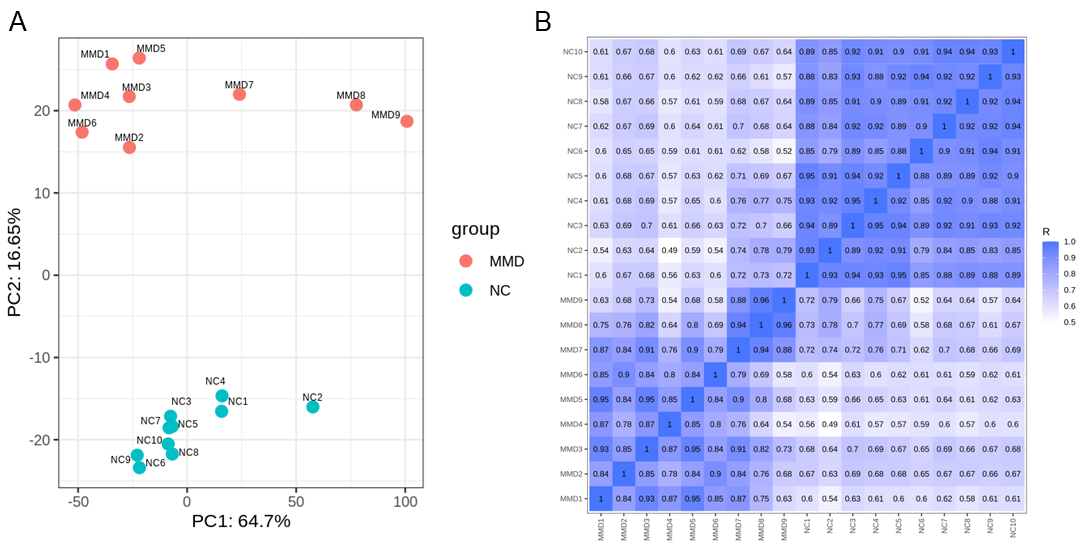
**
