# Supplementary material for: Plasma exosomal microRNAs are non-invasive biomarkers of moyamoya disease: A pilot study
Source: Clinics (Sao Paulo). 2023 Jul 5;78:100247. doi: 10.1016/j.clinsp.2023.100247 (PMC10344806; doi:10.1016/j.clinsp.2023.100247)
Supplement: Supplementary file 2 [file mmc2.docx]

Supplement Figure 1. Principal component diagram (A) and correlation analysis diagram (B) of samples. NC, non-MMD patients. MMD, moyamoya disease.

**
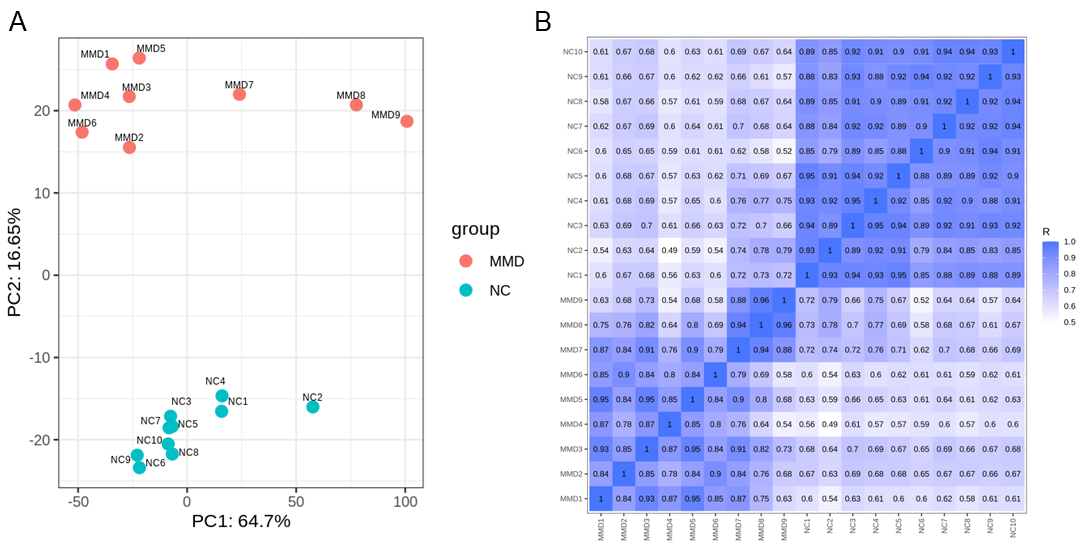
**
